# Genome sequence of the *Lotus spp*. microsymbiont *Mesorhizobium loti* strain R7A

**DOI:** 10.1186/1944-3277-9-6

**Published:** 2014-12-08

**Authors:** Simon Kelly, John Sullivan, Clive Ronson, Rui Tian, Lambert Bräu, Christine Munk, Lynne Goodwin, Cliff Han, Tanja Woyke, Tatiparthi Reddy, Marcel Huntemann, Amrita Pati, Konstantinos Mavromatis, Victor Markowitz, Natalia Ivanova, Nikos Kyrpides, Wayne Reeve

**Affiliations:** 1 Department of Microbiology and Immunology, University of Otago, Dunedin, New Zealand; 2 Centre for Rhizobium Studies, Murdoch University, Perth, Australia; 3 School of Life and Environmental Sciences, Deakin University, Melbourne, Australia; 4 Los Alamos National Laboratory, Bioscience Division, Los Alamos, New Mexico, USA; 5 DOE Joint Genome Institute, Walnut Creek, California, USA; 6 Biological Data Management and Technology Center, Lawrence Berkeley National Laboratory, Berkeley, California, USA; 7 Department of Biological Sciences, King Abdulaziz University, Jeddah, Saudi Arabia

**Keywords:** Root-nodule bacteria, Nitrogen fixation, Symbiosis, *Alphaproteobacteria*

## Abstract

*Mesorhizobium loti* strain R7A was isolated in 1993 in Lammermoor, Otago, New Zealand from a *Lotus corniculatus* root nodule and is a reisolate of the inoculant strain ICMP3153 (NZP2238) used at the site. R7A is an aerobic, Gram-negative, non-spore-forming rod. The symbiotic genes in the strain are carried on a 502-kb integrative and conjugative element known as the symbiosis island or ICE*Ml*Sym^R7A^. *M. loti* is the microsymbiont of the model legume *Lotus japonicus* and strain R7A has been used extensively in studies of the plant-microbe interaction. This report reveals that the genome of *M. loti* strain R7A does not harbor any plasmids and contains a single scaffold of size 6,529,530 bp which encodes 6,323 protein-coding genes and 75 RNA-only encoding genes. This rhizobial genome is one of 100 sequenced as part of the DOE Joint Genome Institute 2010 *Genomic Encyclopedia for Bacteria and Archaea-Root Nodule Bacteria* (GEBA-RNB) project.

## Introduction

*Mesorhizobium loti* strain R7A is a reisolate of strain ICMP3513 (International Culture Collection of Microorganisms from Plants, LandCare Research, Auckland, New Zealand). It was isolated from a root nodule taken from a stand of *Lotus corniculatus* in Lammermoor, Central Otago, New Zealand, inoculated seven years earlier with strain ICMP3153 [1]. Strain ICMP3153 was a recommended inoculant strain for *L. corniculatus* in New Zealand and is also known as NZP2238 and Lc265Da. In its guise as NZP2238, it was one of the strains used to define the species *Rhizobium loti* (now *Mesorhizobium loti*) [2].

Strain R7A contains a 502-kb symbiosis island, also known as ICE*Ml*Sym^R7A^, that was discovered through its ability to transfer from strain ICMP3153 to indigenous nonsymbiotic mesorhizobia at the Lammermoor field site [1,3]. The symbiosis island encodes 414 genes including all of the genes required for Nod factor synthesis, nitrogen fixation and transfer of the island [4]. Transfer of the island occurs via conjugation involving a rolling-circle process. The transferred island integrates into the chromosome of the recipient cell at the sole phenylalanine tRNA gene. Integration of the island is dependent on a P4-type integrase encoded by *intS*, located 198 bp downstream of the phe-tRNA gene, which acts on an attachment site (*attS*) on the circular form of the island and a chromosomal attachment site (*attB*). Integration of the island reconstructs the entire phe-tRNA gene at one end (arbitrarily termed the left end) and forms a 17-bp repeat of the three-prime end of the phe-tRNA gene at the right end of the integrated island [3-5].

*M. loti* is the microsymbiont of the model legume *Lotus japonicus* and strain R7A together with the first *M. loti* strain sequenced, strain MAFF303099 [6], have been used extensively with *L. japonicus* in studies of the plant-microbe interaction. Studies using R7A have included characterization of the symbiotic role of the *vir* Type IV secretion system encoded by the strain [7], determination of the requirements for Nod factor decorations [8] and exopolysaccharides [9] for efficient nodulation of various *Lotus* species, and characterization of genes required for symbiotic nitrogen fixation [10]. The regulation of symbiosis island transfer in strain R7A has also been extensively characterized [11]. Here we present a summary classification and a set of general features for *M. loti* strain R7A together with the description of the complete genome sequence and annotation.

## Classification and general features

**Figure 1:**
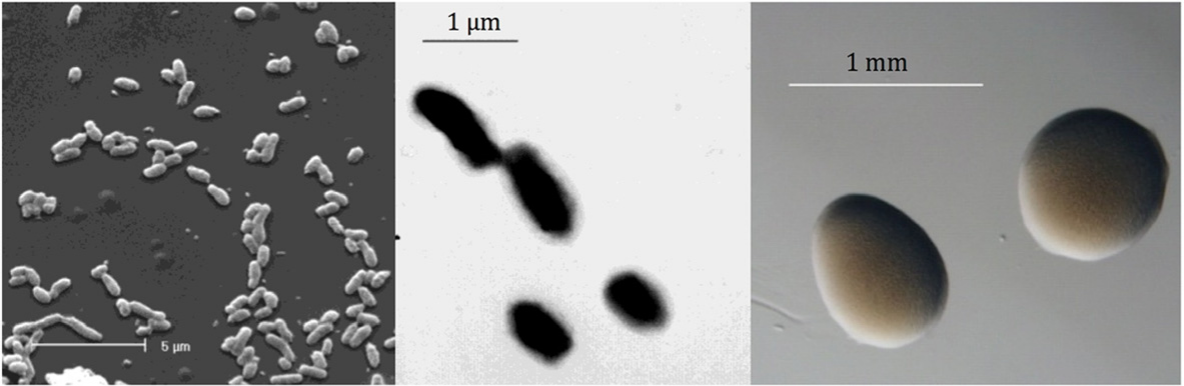
**Images of *Mesorhizobium loti *** **strain R7A using scanning (Left) and transmission (Center) electron microscopy and the appearance of colony morphology on ½LA (Right).**

*Mesorhizobium loti* strain R7A is in the order *Rhizobiales* of the class *Alphaproteobacteria*. Cells are described as non-sporulating, Gram-negative, non-encapsulated, rods. The rod-shaped form varies in size with dimensions of 0.25-0.5 μm in width and 1–1.5 μm in length (Figure 1 Left and 1 Center). They are moderately fast growing, forming 2 mm diameter colonies within 4 days and have a mean generation time of approximately 6 h when grown in TY broth at 28°C [1]. Colonies on G/RDM agar [12] and half strength Lupin Agar (½LA) [13] are opaque, slightly domed, mucoid with smooth margins (Figure 1 Right).

Strains of this organism are able to tolerate a pH range between 4 and 10. Carbon source utilization and fatty acid profiles of *M. loti* have been described previously [2,14,15]. Minimum Information about the Genome Sequence (MIGS) is provided in Table 1.

**Table 1:** Classification and general features of *Mesorhizobium loti* strain R7A according to the MIGS recommendations [16,17]

**MIGS ID**	**Property**	**Term**	**Evidence code**
	Current classification	Domain *Bacteria*	TAS [17]
		Phylum *Proteobacteria*	TAS [18]
		Class *Alphaproteobacteria*	TAS [19]
		Order *Rhizobiales*	TAS [20,21]
		Family *Phyllobacteriaceae*	TAS [21,22]
		Genus *Mesorhizobium*	TAS [14]
		Species *Mesorhizobium loti*	TAS [14]
		Strain R7A	TAS [1]
	Gram stain	Negative	IDA
	Cell shape	Rod	IDA
	Motility	Motile	IDA
	Sporulation	Non-sporulating	NAS
	Temperature range	Mesophile	NAS
	Optimum temperature	28°C	NAS
	Salinity	Unknown	NAS
MIGS-22	Oxygen requirement	Aerobic	TAS [2]
	Carbon source	Various	TAS [23]
	Energy source	Chemoorganotroph	TAS [23]
MIGS-6	Habitat	Soil, root nodule, host	TAS [2]
MIGS-15	Biotic relationship	Free living, Symbiotic	TAS [2]
MIGS-14	Pathogenicity	None	NAS
	Biosafety level	1	TAS [24]
	Isolation	Root nodule of *Lotus corniculatus*	TAS [1]
MIGS-4	Geographic location	Lammermoor, Otago, NZ	TAS [1]
MIGS-5	Nodule collection date	1993	TAS [1]
MIGS-4.1	Latitude	-45.53	TAS [1]
MIGS-4.2	Longitude	169.9415	TAS [1]
MIGS-4.3	Depth	5 cm	IDA
MIGS-4.4	Altitude	885 meters	IDA

Evidence codes – IDA: Inferred from Direct Assay; TAS: Traceable Author Statement (i.e., a direct report exists in the literature); NAS: Non-traceable Author Statement (i.e., not directly observed for the living, isolated sample, but based on a generally accepted property for the species, or anecdotal evidence). These evidence codes are from the Gene Ontology project [25].

**Figure 2:**
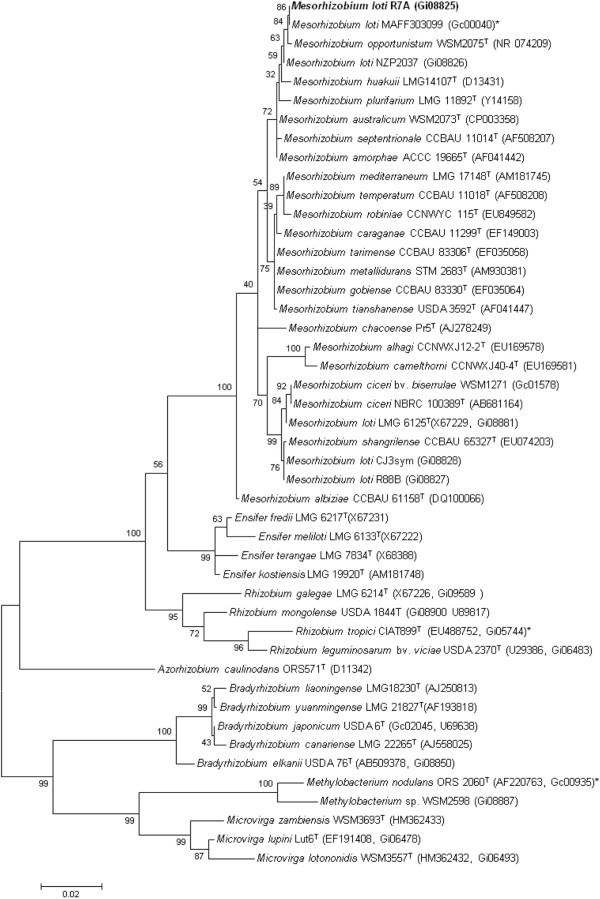
**Shows the phylogenetic neighborhood of *M. loti *strain R7A in a 16S rRNA gene sequence based tree.** This strain has 100% (1,367/1,367 bp) 16S rRNA gene sequence identity to MAFF303099 (GOLD ID: Gc00040) and 99.8% sequence identity (1,364/1,397 bp) to *M. opportunistum* WSM2075 (GOLD ID: Gc01853).

Figure 2 Phylogenetic tree showing the relationships of *Mesorhizobium loti* R7A with other root nodule bacteria based on aligned sequences of the 16S rRNA gene (1,290 bp internal region). All sites were informative and there were no gap-containing sites. Phylogenetic analyses were performed using MEGA [26], version 5. The tree was built using the Maximum-Likelihood method with the General Time Reversible model [27]. Bootstrap analysis [28] with 500 replicates was performed to assess the support of the clusters. Type strains are indicated with a superscript T. Brackets after the strain name contain a DNA database accession number and/or a GOLD ID (beginning with the prefix G) for a sequencing project registered in GOLD [29]. Published genomes are indicated with an asterisk.

## Symbiotaxonomy

*M. loti* strain R7A is a field reisolate of strain ICMP3153 that was originally isolated from a *Lotus corniculatus* nodule in Ireland. It forms effective symbioses with *L. tenuis, L. corniculatus, L. japonicus* (including ecotypes Gifu and MG-20), *L. filicaulis* and *L. burttii.* It also induces but does not infect nodule primordia on *L. pedunculatus* and *Leucaena leucocephala*[7,8]. Mutants of strain R7A defective in the *vir* Type IV secretion system encoded on the symbiosis island are able to form effective nodules on *Leucaena leucocephala* but not *L. pedunculatus*[7]. A nonsymbiotic derivative of R7A cured of the symbiosis island and therefore unable to form root nodules has also been isolated and is called R7ANS [5].

## Genome sequencing and annotation information

### Genome project history

This organism was selected for sequencing on the basis of its environmental and agricultural relevance to issues in global carbon cycling, alternative energy production, and biogeochemical importance, and is part of the Community Sequencing Program at the U.S. Department of Energy, Joint Genome Institute (JGI) for projects of relevance to agency missions. The genome project is deposited in the Genomes OnLine Database [29] and an improved-high-quality-draft genome sequence in IMG. Sequencing, finishing and annotation were performed by the JGI. A summary of the project information is shown in Table 2.

**Table 2:** Genome sequencing project information for *Mesorhizobium loti* R7A

**MIGS ID**	**Property**	**Term**
MIGS-31	Finishing quality	Improved-high-quality-draft
MIGS-28	Libraries used	Illumina Standard (short PE) and CLIP (long PE) libraries
MIGS-29	Sequencing platforms	Illumina HiSeq2000 technology
MIGS-31.2	Sequencing coverage	Illumina: 563×
MIGS-30	Assemblers	Velvet version 1.1.05; Allpaths-LG version r38445 phrap, version 4.24
MIGS-32	Gene calling method	Prodigal 1.4, GenePRIMP
	Genbank accession	AZAM00000000
	Genbank Registration Date	07-FEB-2014
	GOLD ID	Gi08825
	NCBI project ID	74389
	Database: IMG	2512875016
	Project relevance	Symbiotic nitrogen fixation, agriculture

### Growth conditions and DNA isolation

*M. loti* strain R7A was grown to mid logarithmic phase in TY rich medium [30] on a gyratory shaker at 28°C at 250 rpm. DNA was isolated from 60 mL of cells using a CTAB (Cetyl trimethyl ammonium bromide) bacterial genomic DNA isolation method [31].

### Genome sequencing and assembly

The draft genome of *M. loti* R7A was generated at the DOE Joint Genome Institute (JGI) using Illumina data [32]. For this genome, we constructed and sequenced an Illumina short-insert paired-end library with an average insert size of 270 bp which generated 21,315,208 reads and an Illumina long-insert paired-end library with an average insert size of 10487.44 +/- 2154.53 bp which generated 3,077,470 reads totaling 3,659 Mbp of Illumina data (unpublished, Feng Chen). All general aspects of library construction and sequencing performed at the JGI can be found at the DOE Joint Genome Institute website [33].

The initial draft assembly contained 12 contigs in 1 scaffold. The initial draft data was assembled with Allpaths, version 38445, and the consensus was computationally shredded into 10 Kbp overlapping fake reads (shreds). The Illumina draft data were also assembled with Velvet, version 1.1.05 [34], and the consensus sequences were computationally shredded into 1.5 Kbp overlapping fake reads (shreds). The Illumina draft data was assembled again with Velvet using the shreds from the first Velvet assembly to guide the next assembly. The consensus from the second VELVET assembly was shredded into 1.5 Kbp overlapping fake reads. The fake reads from the Allpaths assembly and both Velvet assemblies and a subset of the Illumina CLIP paired-end reads were assembled using parallel phrap, version SPS 4.24 (High Performance Software, LLC). Possible mis-assemblies were corrected with manual editing in Consed [35-37]. Gap closure was accomplished using repeat resolution software (Wei Gu, unpublished), and sequencing of bridging PCR fragments with Sanger technology. A total of 40 additional sequencing reactions were completed to close gaps and to raise the quality of the final sequence. There are 3 contigs and 1 scaffold in the current assembly. The estimated size of the genome is 6.5 Mbp and the final assembly is based on 3,659 Mb of Illumina draft data, which provides an average 563× coverage of the genome.

### Genome annotation

Genes were identified using Prodigal [38] as part of the Oak Ridge National Laboratory genome annotation pipeline, followed by a round of manual curation using the JGI GenePrimp pipeline [39]. The predicted CDSs were translated and used to search the National Center for Biotechnology Information (NCBI) nonredundant database, UniProt, TIGRFam, Pfam, PRIAM, KEGG, COG, and InterPro databases. These data sources were combined to assert a product description for each predicted protein. Non-coding genes and miscellaneous features were predicted using tRNAscan-SE [40], RNAMMer [41], Rfam [42], TMHMM [43], and SignalP [44]. Additional gene prediction analyses and functional annotation were performed within the Integrated Microbial Genomes (IMG-ER) platform [45].

## Genome properties

**Figure 3:**
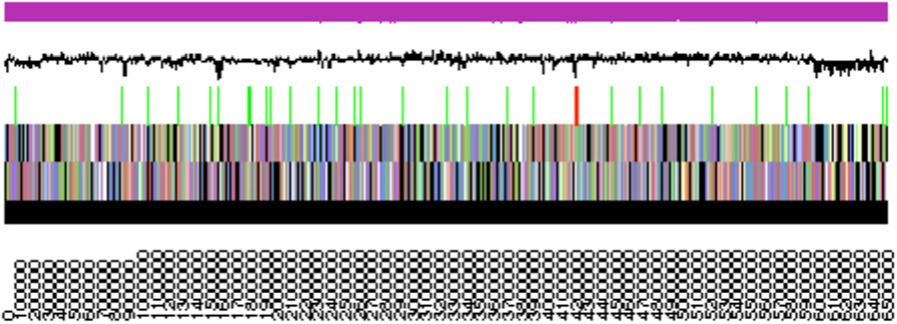
**Graphical map of the single scaffold of *Mesorhizobium loti *R7A.** From bottem to the top: Genes on forward strand (color by COG categories as denoted by the IMG platform), Genes on reverse strand (color by COG categories), RNA genes (tRNAs green, sRNAs red, other RNAs black), GC content, GC skew.

The genome is 6,529,530 nucleotides with 62.93% GC content (Table 3 and Figure 3) and is comprised of a single scaffold and no plasmids. From a total of 6,398 genes, 6,323 were protein encoding and 75 RNA-only encoding genes. Within the genome, 203 pseudogenes were also identified. The majority of genes (80.10%) were assigned a putative function whilst the remaining genes were annotated as hypothetical. The distribution of genes into COGs functional categories is presented in Table 4.

**Table 3:** Genome statistics for *Mesorhizobium loti* R7A

**Attribute**	**Value**	**% of total**
Genome size (bp)	6,529,530	100.00
DNA coding region (bp)	5697197	87.25
DNA G + C content (bp)	4108774	62.93
Number of scaffolds	1	
Number of contigs	3	
Total genes	6,398	100.00
RNA genes	75	1.17
rRNA operons	2*	
Protein-coding genes	6,323	98.83
Genes with function prediction	5,125	80.10
Genes assigned to COGs	5,127	80.13
Genes assigned Pfam domains	5,333	83.35
Genes with signal peptides	565	8.83
Genes coding transmembrane proteins	1,518	23.73

*3 copies of 5S, 2 copies of 16S and 3 copies of 23S rRNA genes.

**Table 4:** **Number of protein coding genes of***Mesorhizobium loti***R7A associated with the general COG functional categories**

**Code**	**Value**	**% age**	**COG category**
J	199	3.49	Translation, ribosomal structure and biogenesis
A	0	0.00	RNA processing and modification
K	521	9.13	Transcription
L	172	3.01	Replication, recombination and repair
B	6	0.11	Chromatin structure and dynamics
D	30	0.53	Cell cycle control, mitosis and meiosis
Y	0	0.00	Nuclear structure
V	65	1.14	Defense mechanisms
T	217	3.80	Signal transduction mechanisms
M	296	5.19	Cell wall/membrane biogenesis
N	53	0.93	Cell motility
Z	0	0.00	Cytoskeleton
W	1	0.02	Extracellular structures
U	124	2.17	Intracellular trafficking and secretion
O	195	3.42	Posttranslational modification, protein turnover, chaperones
C	304	5.33	Energy production conversion
G	511	8.95	Carbohydrate transport and metabolism
E	675	11.83	Amino acid transport metabolism
F	89	1.56	Nucleotide transport and metabolism
H	216	3.78	Coenzyme transport and metabolism
I	242	4.24	Lipid transport and metabolism
P	249	4.36	Inorganic ion transport and metabolism
Q	181	3.17	Secondary metabolite biosynthesis, transport and catabolism
R	750	13.14	General function prediction only
S	612	10.72	Function unknown
-	1,271	19.87	Not in COGS

## Conclusions

The *M. loti* R7A genome consists of a single 6.5-Mb chromosome which encodes 6,398 genes. The sequencing was completed to the stage where a single scaffold comprising 3 contigs was obtained. *M. loti* strain R7A and *M. loti* strain MAFF303099 are currently the two most widely studied *M. loti* strains. Strain R7A differs from MAFF303099 in that the genome lacks plasmids whereas the genome of MAFF303099 includes two plasmids pMLa and pMLb [6]. The R7A symbiosis island remains mobile whereas the MAFF303099 symbiosis island is likely immobile due at least in part to a transposon insertion within the origin of transfer (*oriT*) [3,5]. *M. loti* strain R7A represents an important resource for the study of the mechanism and regulation of transfer of large mobile integrative and conjugative elements (ICEs). It is also widely used in conjunction with the model legume *Lotus japonicus* for ongoing molecular analyses of the plant-microbe interactions required for the establishment of a nitrogen-fixing symbiosis.

## Competing interests

The authors declare that they have no competing interests.

## Authors’ contributions

JS and CR supplied the strain and background information for this project and helped WR write the paper, TR supplied DNA to JGI and performed all imaging, WR coordinated the project and all other authors were involved in either sequencing the genome and/or editing the paper. All authors read and approved the final manuscript.
